# Reduced Pelvic Floor Muscle Tone Predisposes to Persistence of Lower Urinary Tract Symptoms after Puerperium

**DOI:** 10.1155/2016/5705186

**Published:** 2016-03-28

**Authors:** Chandana Bhat, Mahjabeen Khan, Kirthinath Ballala, Asha Kamath, Deeksha Pandey

**Affiliations:** KMC Manipal, Manipal University, Manipal, Karnataka 576104, India

## Abstract

Pregnant primiparous women at term were enrolled in the study. ICIQ-FLUTS questionnaire was used to find out prevalence of LUTS. MOS was used to assess pelvic floor muscle strength. Women were followed up after 8–10 weeks of delivery to find out remission or persistence of these symptoms. We found that increased frequency of micturition was the most common (82%) LUTS seen in primiparous women at term. More than half (51%) of these women who complained of LUTS had a poor pelvic floor muscle tone (MOS grade 3). Out of those who had symptoms during pregnancy 11% remained symptomatic even after puerperium. Interestingly 61% of those with persistence of symptoms demonstrated a very poor pelvic floor muscle tone at term (MOS grade 2), while the remaining 39% also had a tone of only MOS grade 3. Thus women with LUTS during pregnancy should be screened for their pelvic floor muscle tone with simple MOS system which will help to predict the persistence of these symptoms later on. Women with a low score (three or less) should be triaged for regular pelvic floor muscle exercises.

## 1. Introduction

Many women develop Lower Urinary Tract Symptoms (LUTS) during pregnancy. LUTS involves problems related to storage and voiding of urine. Most of these symptoms are physiological and revert back to normal after puerperium. However in a fraction of women these symptoms may persist. Urinary incontinence that starts during pregnancy and postpartum has been postulated as a significant risk factor for urinary incontinence later in life [1]. Multiple theories have been postulated to explain the onset of LUTS during pregnancy. What causes these symptoms to persist in some women remains a dilemma. Investigators tried to correlate persistence of these symptoms with the mode of delivery. Symptoms other than stress urinary incontinence (SUI) that include urgency, frequency, and nocturia that are triggered during pregnancy were found to have no relation to the mode of delivery [2].

Pelvic floor muscles have a fundamental role in supporting the pelvic organs and facilitating their normal functioning. During pregnancy this supporting structure is overloaded because of the progressive growth of the uterus with the developing foetus inside it [3]. Change in the hormonal milieu during pregnancy also contributes to the change in tissue structure leading to alterations in the tone and functioning of pelvic structures [4, 5].

In the present study we hypothesized that pelvic floor muscle tone during pregnancy has a role to play in the LUTS. The study was aimed at evaluating association between pelvic floor muscle tone at term and presence or persistence of LUTS in primiparous women.

## 2. Material and Methods

This cross-sectional study was conducted at a tertiary care centre affiliated to a medical university. The project followed ethical guidelines of the Institutional Review Board and was approved by Ethics Committee, Kasturba Hospital Manipal, Karnataka, India (IEC 569/2014). Pregnant primiparous women who were admitted at term for delivery from January 2014 to May 2015 were enrolled in the study. Those with multiple gestation, placenta previa, and intrauterine death were excluded. Women who gave history of LUTS prior to pregnancy were also excluded. All women as a routine protocol underwent microscopic examination of urine, which was followed by confirmation of infection by urine culture. Women who had evidence of infection on urine culture too were excluded from the study.

All participants were provided with a study information sheet and they were allowed to ask questions regarding the study and their participation. A written informed consent was then obtained. The first step of the study was to determine the prevalence of LUTS during pregnancy at term. Thus, as recommended by ICS that every epidemiological study about urinary incontinence should include standardized ICS questionnaires, we included the International Consultation on Incontinence Questionnaire on Female Lower Urinary Tract Symptoms (ICIQ-FLUTS) in our study. This questionnaire includes 13 questions related to LUTS experienced within the 4 weeks preceding the survey, to which responses are made on a 5-category (0–4) scale depending on presence of symptom and perceived severity.

An independent observer who was blinded to the responses in the questionnaire then performed a digital introital examination to determine the tone of pelvic floor musculature. Observations thus obtained were graded according to the standardized Modified Oxford Score (MOS) system (score 0–5). Zero here meant absence of muscle contraction; one meant flicker of muscles; two meant week contraction; three meant medium contraction—slight lift of examiner's finger without resistance; four meant strong contraction—elevation of examiner's finger against small resistance; and five meant very strong contraction—elevation of examiners finger against strong resistance. Findings were recorded and then correlated with the LUTS based on the ICIQ-FLUTS questionnaire. Those women who reported LUTS during pregnancy in this group were followed up after puerperium (8–10 weeks following delivery). LUTS were then reassessed with the same ICIQ-FLUTS questionnaire to find out the resolution/persistence of those symptoms which had developed de novo during pregnancy.

### 2.1. Sample Size Calculation

The sample size was determined as a minimum of 269 study subjects considering an anticipated prevalence of 46% at 90% confidence level and 5% absolute precision.

### 2.2. Statistical Analysis

All calculations were made using SPSS 21.0 IBM Statistics released August 2012 (IBM Corporation 1, New Orchard Road, Armonk, NY 10504-1722, USA). Categorical variables were compared using the chi square test. Significance was set at *p* < 0.05.

## 3. Results

**Figure 1:**
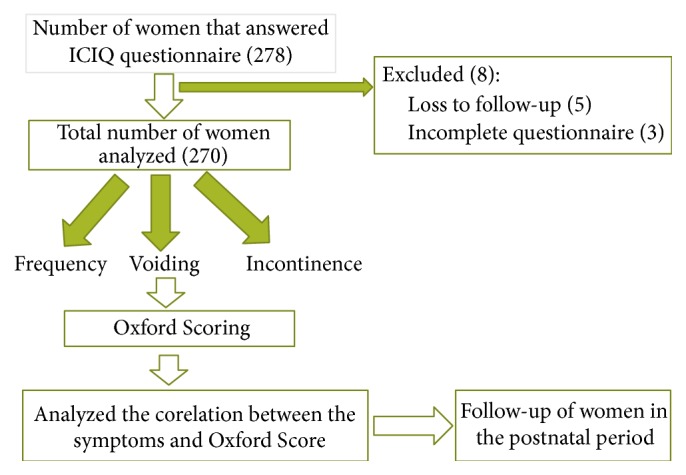
Consort statement.

A total of 278 women consented to participate in the study. However 3 questionnaires were incomplete and thus excluded from the final analysis. Five women were lost to follow-up after delivery and could not be contacted. Thus a total of 8 women were excluded, making the final number 270 for the analysis (Figure 1).

### 3.1. Demographic Characteristics

Mean age of the cohort studied was 26 years. All women included in the study were primiparous as per the inclusion criteria. Mean gestational age was 37 weeks. On comparing the patients who were found to have LUTS to those who did not have it, the symptoms in both the groups were found to be demographically comparable (Table 1).

### 3.2. LUTS at Term

**Figure 2:**
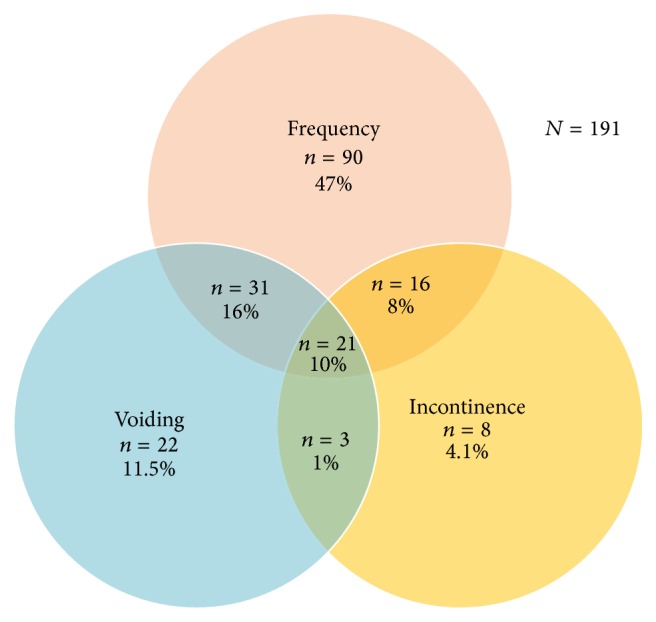
Distribution of various LUTS in the study population (we found a considerable overlap between the 3 main groups of symptoms).

On analyzing the symptoms based on the filled ICIQ-FLUTS questionnaire, 70.8% (*n* = 191) of women were found to be having some or the other LUTS. Out of these symptomatic women 90 (47%) had frequency and 22 (11.5%) had voiding problems, while only 8 (4.1%) complained of incontinence. Certain degree of overlap was also found among these symptoms (Figure 2). A total of 31 women (10%) had both frequency and voiding difficulties, 8 women (16%) complained of frequency of urination as well as incontinence, and 3 (1%) had frequency with incontinence.

**Figure 3:**
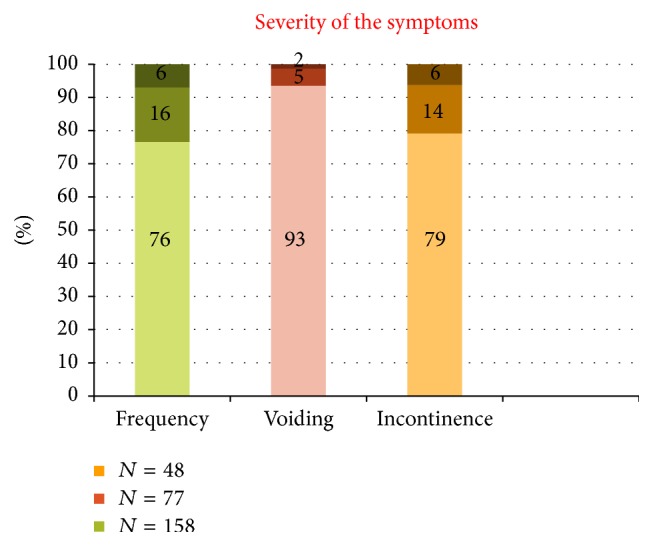
Severity grading of various LUTS.

For the symptomatic women, however, when asked to grade their severity of these symptoms, majority reported it as mild (Figure 3). Out of 158 women complaining of frequency 76% described the symptom to be not causing much inconvenience, and 93% of 77 women with voiding symptoms reported it to be mild and so did 79% of 48 women having incontinence.

### 3.3. Correlation of LUTS and Pelvic Floor Muscle Tone

On comparing the pelvic floor muscle strength of symptomatic versus asymptomatic women, we found a good correlation between the two (Table 2). Most of the women who had LUTS had the muscle tone of grades 3 and 4 on MOS while most women who did not have any LUTS had muscle tone of grade 5 on MOS.

### 3.4. Persistence of Symptoms after Puerperium

On following up patients who had LUTS at term we found out that in 10.9% of women (*n* = 21) symptoms persisted. Frequency of micturition was the most common symptom at term as well as the most common symptom to persist after puerperium, followed by voiding difficulties and incontinence (Table 3). No patient in the LUTS group developed de novo symptoms after puerperium. On comparing various characteristics like age, gestational age at the time of delivery, BMI, labour duration, birth weight, and medical and obstetric complication both the groups (with or without LUTS) were similar (Table 4).

When we looked back into the data of these women who had persistence of symptoms, it was obvious that these women had a lower grade of pelvic floor muscle tone [2, 3] as compared to the women who recovered (Table 5).

## 4. Discussion

Many women suffer from LUTS during pregnancy. A number of pregnancy related changes have been found to be responsible for these symptoms [6]. The more interesting observation is that for some women these symptoms resolve with puerperal healing while in some women it persists. The reason for this persistence in some women remains unclear. Pregnancy and child birth are known to affect pelvic floor muscle strength, causing LUTS and affecting the quality of life [7, 8].

In comparison to the data from the Western countries we found a lower prevalence (only 8%) of urinary incontinence in our population [7, 9]. Among the Southeast Asian countries the prevalence of urinary incontinence has been found to be between 26% and 46% [10–12]. In India, the prevalence of stress and mixed and urge incontinence during pregnancy was reported to be 19.2%, 3.8%, and 2.9%, respectively [13]. The plausible reason for this low incidence in our cohort might be the inclusion of only primiparous women in our study cohort. Moreover in other studies patients having preexistent urinary incontinence or other LUTS were not excluded [14].

In the present study we found that increased frequency of micturition was the most common (82%) LUTS observed among primiparous women at term. Similar to our observation other studies also have found that frequency of micturition is the most common LUTS during pregnancy [10, 14].

The severity of the symptoms reported by the women was mild. The study showed that 76%, 93%, and 79% of women with frequency, voiding, and incontinence symptoms, respectively, described their symptoms as not very bothersome. In 191 women experiencing LUTS 14 of them complained of nocturia.

We used MOS to determine the pelvic floor muscle strength, as it has already been proved that MOS done by digital examination is a valid method for assessment of pelvic floor muscle strength. Studies have found it to be comparable to perineometry [15–18]. More than half (55%) of these women who complained of LUTS had a poor pelvic floor muscle tone (MOS grade 3 or less).

Contrary to our findings, study done in 91 primigravid women at 30–34 weeks of pregnancy could not find any association between LUTS and pelvic floor muscle strength [19]. This difference in findings might be because of a difference in the timing of observation at two different periods of gestation. During pregnancy, pelvic floor muscles strength decreases from 14th week onwards, owing to the increasing levels of circulating relaxin. After the 24th week, the levels of relaxin stabilize and remain constant till the end of pregnancy [20]. In later gestation increased weight of gravid uterus coupled with higher levels of progesterone and continuous alterations in collagen microsculpture may cause decrease in muscle tone. More studies are required to determine whether or not pelvic floor muscle tone contributes to LUTS, as this is one of the easily modifiable factors with exercise.

In our cohort, we did not find any case of de novo LUTS after puerperium, implying that women who were asymptomatic in pregnancy remained so even after puerperium. Out of those who had symptoms during pregnancy only a small proportion of women (11%) remained symptomatic even after puerperium. Interestingly 61% of those with persistence of symptoms demonstrated a very poor pelvic floor muscle tone at term (MOS grade 2), while the remaining 39% also had a tone of only MOS grade 3.

In this study we did not concentrate on the role of mode of delivery in de novo LUTS following delivery as it has already been proved that mode of delivery contributes significantly to the de novo occurrence of these symptoms [21–23].

We followed up women only up to 8–10 weeks after delivery. A longer follow-up for persistence or resolution of these symptoms could yield more conclusions.

To our knowledge, our study is the first to demonstrate an easily modifiable factor that can lead to persistence of LUTS after the remodelling effects of puerperium are over. Thus, we emphasize the need to screen women during pregnancy for reduced pelvic floor muscle strength by a simple digital examination and to triage these women for pelvic floor muscle exercises.

## 5. Conclusion

Women with LUTS during pregnancy should be screened for their pelvic floor muscle tone with simple MOS system which will help to predict the persistence of these symptoms later on. Women with a low score (three or less) should be triaged for regular pelvic floor muscle exercises.

## Figures and Tables

**Table 1:** Demographic characteristics of the study population.

Characteristics	LUTS present (191)	LUTS absent (79)	*p* value
Mean age ± SD	26.53 ± 3.69	26.81 ± 4.467	0.60
Mean gestational age ± SD	37.61 ± 0.13	38.16 ± 1.247	0.50
Medical disorder complicating pregnancy: *n* (%)	08 (07.8)	05 (06.3)	0.50
Obstetric complications: *n* (%)	17 (08.9)	11 (10.1)	0.50
Mean Body Mass Index (BMI) ± SD	21.72 ± 1.38	21.80 ± 1.45	0.66

**Table 2:** Comparison of patients with or without LUTS with reference to the pelvic floor muscle tone.

Modified Oxford Score (MOS)	LUTS present *N* (%)	LUTS absent *N* (%)
2	07 (03)	00
3	98 (51)	04 (02)
4	56 (29)	15 (19)
5	30 (17)	60 (79)
Total	191 (100)	79 (100)

**Table 3:** Persistence of symptoms after puerperium.

LUTS	Frequency *N* (%)	Voiding problems *N* (%)	Incontinence *N* (%)
At term	158 (82)	77 (40)	48 (25)
After puerperium (21)	02 (10)	02 (10)	17 (80)

**Table 4:** Comparison of characteristics which might affect persistance or resolution of LUTS after puerperium.

Characteristics	LUTS present (21)	LUTS absent (249)	*p* value
Mean age ± SD	27.57 ± 4.99	26.53 ± 3.82	0.24
Mean gestational age ± SD	37.81 ± 2.05	37.77 ± 1.92	0.92
Medical disorder complicating pregnancy: *n* (%)	02 (09.5)	18 (07.22)	
Obstetric complications: *n* (%)	03 (14.2)	22 (08.8)	
Mean Body Mass Index (BMI) ± SD	21.69 ± 1.04	21.77 ± 1.43	0.80
Mean duration of labour (minutes) ± SD	360 ± 68	331.3 ± 129	0.31
Mean birth weight ± SD	2580 ± 266.4	2722.8 ± 348.2	0.07

**Table 5:** Pelvic floor muscle tone (during pregnancy) of women who had persistent LUTS after puerperium.

	Frequency *N* = 2	Voiding problems *N* = 2	Incontinence *N* = 17
MOS, 2	02	02	12 (54.5%)
MOS, 3	00	00	05 (22.7%)
